# Endobronchial Extension of Metastatic Osteosarcoma Causing Central Airway Obstruction: A Case Report

**DOI:** 10.1155/cris/8255987

**Published:** 2026-10-21

**Authors:** Mona Dasgupta, Jesse Woodall, Whitney Brinkley, Christina Glenn, Christian Probst, Paul Branca, Benjamin Bevill, Sean Jordan

**Affiliations:** ^1^ Division of Pulmonary and Critical Care Medicine Department of Internal Medicine University of Tennessee Medical Center Knoxville Tennessee USA tennessee.edu; ^2^ Department of Internal Medicine University of Tennessee Medical Center Knoxville Tennessee USA tennessee.edu; ^3^ Department of Pathology University of Tennessee Medical Center Knoxville Tennessee USA tennessee.edu; ^4^ Division of Thoracic Surgery Department of Surgery University of Tennessee Medical Center Knoxville Tennessee USA tennessee.edu

## Abstract

Metastatic osteosarcoma most commonly involves the lungs and typically presents as peripheral pulmonary nodules amenable to limited resection. Endobronchial extension of metastatic osteosarcoma resulting in critical central airway obstruction, however, is rare. We present a woman in her late 60s with a history of distal femoral osteosarcoma treated 15 years earlier who developed progressive respiratory symptoms initially treated as pneumonia. Imaging demonstrated a large right‐sided endobronchial mass causing obstruction of the right mainstem bronchus and impending compromise of the left mainstem bronchus. Urgent flexible bronchoscopy was performed with rigid bronchoscopy and extracorporeal membrane oxygenation available on standby as rescue measures. Tumor debulking using hot snare resection and forceps restored ventilation to the left lung and stabilized the airway. Because of extensive hilar and endobronchial involvement, definitive surgical management required right pneumonectomy via a serratus‐sparing posterolateral thoracotomy. Pathologic examination demonstrated a 14.5‐cm metastatic osteosarcoma originating in the right middle lobe with negative bronchial, vascular, and parenchymal margins and no lymph node involvement. The patient recovered without major complications. This case highlights a rare presentation of metastatic osteosarcoma causing life‐threatening central airway obstruction and demonstrates the importance of multidisciplinary management, bronchoscopic airway stabilization, and definitive surgical resection in carefully selected patients.

**Summary**

• Early recognition of endobronchial metastasis: in patients with a history of osteosarcoma and new or progressive respiratory symptoms, endobronchial metastatic disease should be considered, particularly when imaging demonstrates a central airway mass or obstruction.
• Multidisciplinary airway stabilization: life‐threatening central airway obstruction may require coordinated management among interventional pulmonology, thoracic surgery, anesthesia, and critical care, with bronchoscopic tumor debulking serving as a bridge to definitive treatment.
• Careful patient selection for pneumonectomy: although pulmonary metastasectomy generally favors parenchyma‐sparing approaches, pneumonectomy may be appropriate in carefully selected patients with extensive central or hilar disease when complete resection is feasible and multidisciplinary evaluation supports surgical candidacy.

## 1. Introduction

Endobronchial metastases from osteosarcoma are rare as most pulmonary metastases arise in the peripheral parenchyma as pulmonary nodules [1, 2] and can be removed with wedge excision. Kiryu et al. [3] classified endobronchial metastases from nonpulmonary neoplasms into four categories as follows: type I, direct bronchus metastasis, type II, bronchial invasion via a parenchymal lesion, type III, bronchial invasion via mediastinal or hilar lymph nodes, and type IV, peripheral lesions extending along a proximal bronchus. In their study, Kiryu et al. [3] noted that a type II lesion was found in 1/16 patients compared to the other types, highlighting the rarity of this form.

In this case, a parenchymal metastasis originating in the right middle lobe developed a large luminal polypoid component that extended into and occluded the right mainstem bronchus. This pattern is consistent with a Kiryu type II endobronchial metastasis, in which a parenchymal lesion invades the bronchial lumen [3], resulting here in acute symptomatic central airway obstruction. Although pulmonary metastases from osteosarcoma are common, endobronchial metastases causing life‐threatening central airway obstruction are uncommon. Furthermore, most pulmonary metastases are amenable to wedge resection, whereas this patient ultimately required pneumonectomy because of extensive hilar and endobronchial involvement. Here, we report a case of isolated pulmonary metastatic osteosarcoma presenting with life‐threatening central airway obstruction requiring bronchoscopic tumor debulking and subsequent open pneumonectomy.

## 2. Case Report

A woman in her 60s was transferred from an outside hospital for an endobronchial mass associated with rapidly progressive airway compromise. She had been treated empirically for pneumonia for several months before eventually undergoing computed tomography (CT) of the chest, which revealed a large right lung mass (Figure 1). On arrival, she required 2 L nasal cannula oxygen to maintain a saturation of 92%. A PET scan performed a few weeks prior showed a right lower lobe moss‐like density extending into the right airway centrally with a max SUV of 11.6 with no evidence of regional nodal or distant metastatic disease.

**Figure 1:**
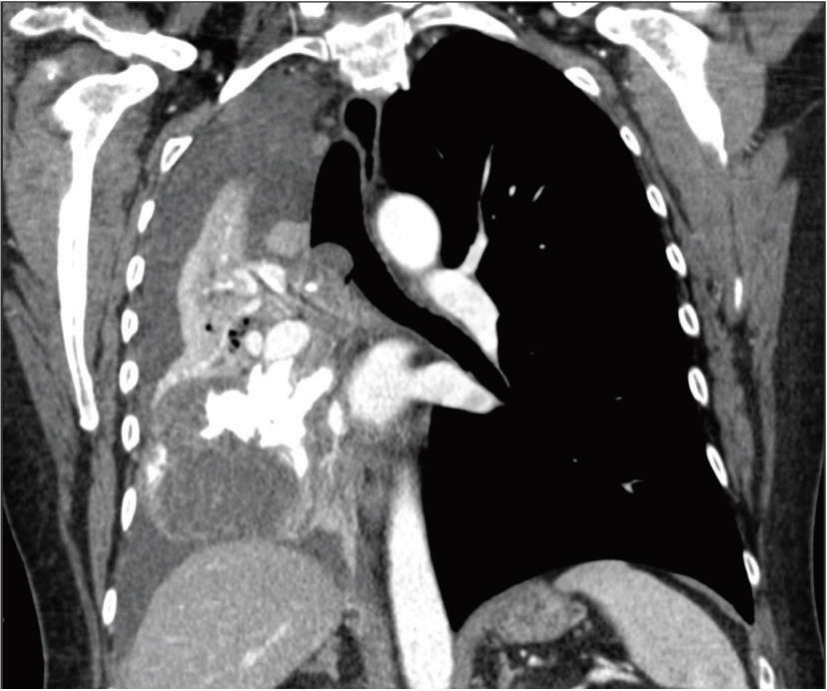
CT chest revealing a large right lung mass.

Interventional pulmonology performed urgent flexible bronchoscopy for tumor debulking, with rigid bronchoscopy and ECMO on standby given the concern for potential airway loss. Bronchoscopy revealed the tumor completely obstructing the right mainstem bronchus and protruding across the main carina causing partial obstruction of the distal trachea and left mainstem bronchus. The left mainstem had a tumor protruding across it, narrowing it by ~80% endobronchially. Debulking was performed using snare resection and forceps. Jet ventilation was utilized during snare excision due to the risk that dislodged tumor fragments could occlude the left mainstem bronchus. The tumor was debrided to the level of the proximal right mainstem bronchus to preserve the patency of the left mainstem bronchus. Due to the pedunculated nature of the mass, the decision to use a hot snare was made, hoping this would allow for resection of large amounts of the mass while ensuring airway patency. Although the lesion appeared more like soft tissue during endobronchial evaluation, significant difficulty was encountered during hot snare resection as the snare was unable to transect the tumor despite optimization of electrocautery settings. This was suspected to be secondary to calcific deposition related to the patient’s known osteosarcoma, resulting in minimal soft tissue for effective electrocautery. The decision was made to switch to mechanical debulking with the snare, which was ultimately successful, although substantial resistance was encountered.

The procedure was performed using flexible bronchoscopy alone. Rigid bronchoscopy was not utilized as there was minimal bleeding, and it was not felt to provide additional procedural benefit. In addition, there was no clear plane to core out the tumor because the lesion did not appear adherent to the right mainstem bronchial wall and instead appeared to emanate from the right lower lobe.

Overall, the bronchoscopic intervention successfully achieved the primary goal of maintaining ventilation to the left lung, while the right lung remained extensively involved by the tumor.

Histopathology demonstrated a high‐grade spindle cell neoplasm with malignant osteoid, consistent with metastatic osteosarcoma (Figure 2). The patient reported a history of localized distal left femur osteosarcoma treated with chemotherapy and surgical resection in 2011. Given her remote history of distal femoral osteosarcoma, the lesion was interpreted as metastatic osteosarcoma. Original pathology records from the 2011 tumor were unavailable for comparison. She reported that after her initial diagnosis of distal left femur osteosarcoma, she had undergone annual CT surveillance for 5 years. Then, 4 months prior to presenting at our facility, she developed respiratory symptoms, for which her primary care physician ordered chest radiographs. These radiographs suggested she had pneumonia, for which she received antibiotics; however, her symptoms continued, prompting a chest CT that showed an endobronchial lesion.

**Figure 2:**
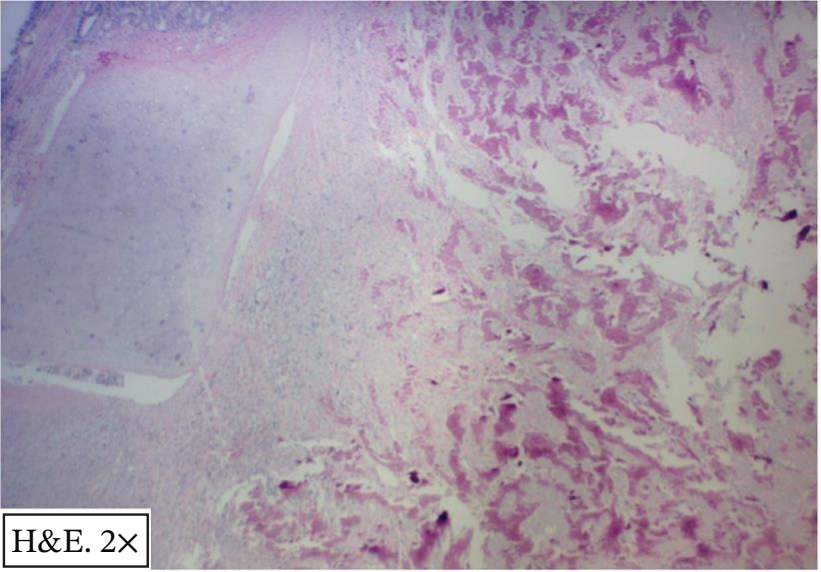
Osteosarcoma with atypical spindle cells and osteoid matrix production. H&E, 2× magnification.

Surgical resection was deemed the only viable option to prevent fatal airway obstruction. Preoperative PET imaging suggested the patency of the right upper lobe bronchus, and a right middle and lower bilobectomy or pneumonectomy was planned. Intraoperatively, however, tumor extension across the fissures precluded the preservation of the right upper lobe, necessitating a right pneumonectomy. A serratus‐sparing posterolateral thoracotomy was performed.

Dissection was difficult due to the extensive tumor burden limiting lung mobilization. The pulmonary veins were individually dissected and divided, followed by the stapled division of the main pulmonary artery. The right mainstem bronchus was then opened sharply, and the intraluminal tumor—extending proximally but without evidence of invasion—was removed. The right mainstem bronchus was stapled at the level of the carina. A pedicled intercostal muscle flap was harvested and used to buttress the bronchial stump.

The patient tolerated the procedure well. Postoperative pathology revealed a 14.5 cm metastatic osteosarcoma originating in the right middle lobe (Figure 3). Lymph nodes and all margins, including bronchial and vascular, were all negative. Preoperative imaging and bronchoscopic evaluation suggested that the tumor arose from the right lower lobe, with extensive extension into the bronchus intermedius and right mainstem bronchus. However, following pneumonectomy, pathologic examination demonstrated that the primary pulmonary tumor originated in the right middle lobe. The discrepancy was likely related to the extensive tumor burden and transfissural extension, with the bulky right middle lobe tumor extending inferiorly toward the right lower lobe and centrally into the bronchus intermedius.

**Figure 3:**
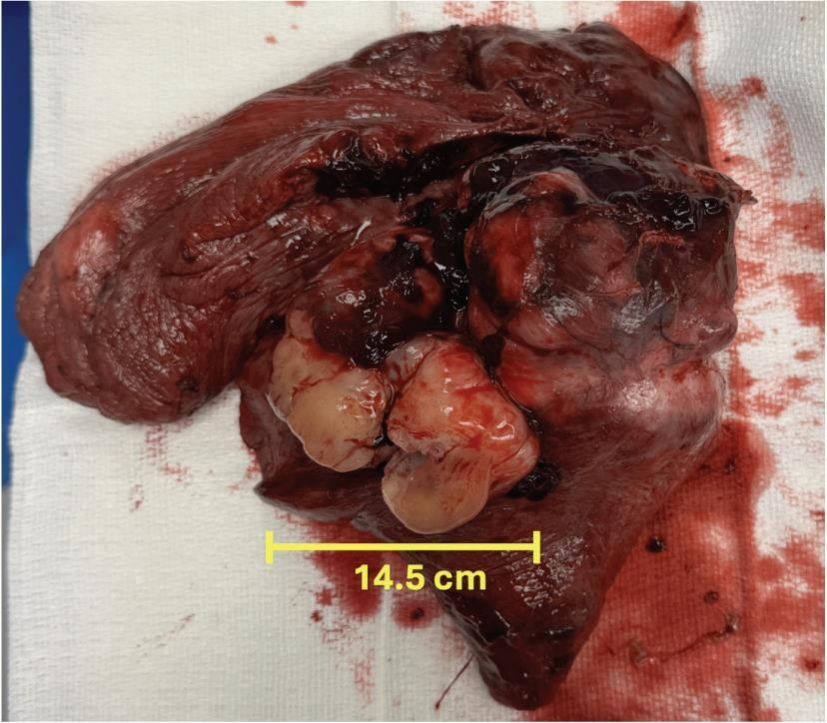
Gross specimen demonstrating a 14.5‐cm metastatic osteosarcoma after pneumonectomy, illustrating the large tumor burden not fully appreciated on bronchoscopic evaluation.

The patient began ifosfamide and etoposide, which were discontinued due to neurotoxicity. The patient established care with medical oncology to continue surveillance imaging every 3 months. Nine months after surgical resection, the patient was noted to have multiple pulmonary nodules on CT thorax, concerning metastatic disease. The patient initially declined therapy with regorafenib, a multikinase inhibitor, and opted to continue surveillance. Another 3 months passed, and she was found to have peritoneal metastasis, at which point she agreed to start regorafenib. Unfortunately, the patient continued to have evidence of metastatic growth, and the decision was made to discontinue regorafenib, and the patient is discussing the risks and benefits of starting systemic chemotherapy with either gemcitabine and docetaxel or gemcitabine monotherapy.

## 3. Discussion

Central endobronchial osteosarcoma presents unique diagnostic and surgical challenges. In the differential diagnosis of a calcified endobronchial mass, metastatic osteosarcoma should be distinguished from primary osseous or cartilaginous airway tumors based on the clinical history, radiographic pattern, and histopathologic findings. Bronchoscopic debulking enabled emergent airway stabilization and facilitated definitive surgical planning [4]. Bulky tumor burden and associated hilar inflammation complicated intraoperative dissection, necessitating meticulous vascular control. A serratus‐sparing posterolateral thoracotomy—more commonly used in lobectomy or limited resections—offered excellent exposure while minimizing postoperative pain and preserving shoulder function. A pedicled intercostal muscle flap was used to reinforce the bronchial stump and reduce the risk of bronchopleural fistula after pneumonectomy [5].

Osteosarcoma metastasizes to the lungs in 10%–17% of patients at diagnosis [6–8], and 20%–33% develop pulmonary metastases over their disease course. Surgeons commonly perform pulmonary metastasectomy, where they remove the tumor in the lungs, in select patients with metastatic solid tumor disease [9, 10]. The preferred method is generally through wedge resections or excision by electrocautery or laser ablation of peripheral lesions [11]. Pneumonectomy for such patients occurs in 1% of these patients as it is reserved for central lesions and carefully selected patients with multidisciplinary management [11] and has been previously reported in three patients with metastatic osteosarcoma. Attar et al. [12] reported two patients with centrally located metastatic osteosarcoma requiring pneumonectomy, while Chang et al. [13] described a patient undergoing completion pneumonectomy for recurrent hilar metastatic disease. Similar to these reports, our patient exhibited extensive central and hilar involvement, necessitating pneumonectomy. However, unlike prior reports, our patient presented with near‐complete central airway obstruction, requiring urgent bronchoscopic debulking before definitive surgical resection.

Surgical resection is often followed by chemotherapy, even in the setting of pneumonectomy. The standard chemotherapy regimen for osteosarcoma consists of doxorubicin, cisplatin, and high‐dose methotrexate [14]. Prophylactic whole lung irradiation can be used postoperatively but is not effective without systemic chemotherapy [15, 16]. When the disease becomes metastatic, the addition of etoposide and high‐dose ifosfamide chemotherapy agents can be used [17], as seen in our patient.

Although endobronchial metastases from osteosarcoma are rare, the pattern of airway involvement in our case is consistent with the recognized Kiryu et al. [3] type II growth behavior in which the parenchymal tumor gave rise to a large tumor that extended proximally into the bronchus and right mainstem bronchus. In our case, the unusual feature was not the direction of tumor growth into the airway lumen but rather the extensive proximal extension resulting in near‐complete central airway obstruction and the need for urgent bronchoscopic stabilization followed by pneumonectomy.

Recent studies suggest that interactions between metastatic osteosarcoma cells and the pulmonary microenvironment play a critical role in facilitating lung colonization and progression. Several molecular pathways may contribute to the enhanced capacity of osteosarcoma to migrate to the lungs [14]. Matrix metalloproteinases, particularly MMP‐2 and MMP‐9, facilitate invasion and have been associated with pulmonary metastasis [14]. Recent single‐cell sequencing studies have also identified alterations in immune checkpoint signaling and CD8+ T‐cell function within pulmonary metastases, highlighting potential future therapeutic targets [18].

What distinguishes this case is the patient’s rapidly progressive central airway obstruction requiring both bronchoscopic tumor debulking and subsequent open pneumonectomy, an approach rarely reported in metastatic osteosarcoma. This case underscores the importance of a multidisciplinary approach in managing rare central airway tumors and demonstrates that even years after initial osteosarcoma treatment, late pulmonary metastases can present with life‐threatening airway compromise.

## Funding

No funding was received for this study.

## Ethics Statement

This case report was reviewed by the University of Tennessee College of Medicine–Knoxville Institutional Review Board and determined to be a case report that does not meet the definition of human subject research under 45 CFR 46. The IRB provided acknowledgment and approval of the case report on July 14, 2026 (Case Report CR#703). The case report was conducted in accordance with the ethical principles of the Declaration of Helsinki.

## Consent

The patient provided informed consent for the use and publication of her health information.

## Conflicts of Interest

The authors declare no conflicts of interest.

## Data Availability

The data that support the findings of this study are available upon request from the corresponding author. The data are not publicly available due to privacy or ethical restrictions.
